# Biochemical Characterization of the Amylase Activity from the New Haloarchaeal Strain *Haloarcula* sp. HS Isolated in the Odiel Marshlands

**DOI:** 10.3390/biology10040337

**Published:** 2021-04-16

**Authors:** Patricia Gómez-Villegas, Javier Vigara, Luis Romero, Cecilia Gotor, Sara Raposo, Brígida Gonçalves, Rosa Léon

**Affiliations:** 1 Laboratory of Biochemistry, Department of Chemistry, Marine International Campus of Excellence (CEIMAR), University of Huelva, Avda. de las Fuerzas Armadas s/n, 21071 Huelva, Spain; patgomvil@gmail.com (P.G.-V.); vigara@uhu.es (J.V.); 2 Instituto de Bioquímica Vegetal y Fotosíntesis, Consejo Superior de Investigaciones Científicas and Universidad de Sevilla, Avenida Américo Vespucio 49, 41092 Seville, Spain; lromero@ibvf.csic.es (L.R.); gotor@ibvf.csic.es (C.G.); 3 CIMA—Centre for Marine and Environmental Research, FCT, Campus de Gambelas, Universidade do Algarve, 8005-139 Faro, Portugal; sraposo@ualg.pt (S.R.); bgrodrigues@ualg.pt (B.G.)

**Keywords:** amylase, extremozymes, haloarchaea, enzymatic characterization, proteomics

## Abstract

**Simple Summary:**

Amylases are a group of enzymes that degrade starch into simple sugars. These proteins are produced by a wide variety of organisms and are supposed to be one of the most valuable industrial enzymes. However, the extreme conditions required for many industrial operations limit the applicability of most amylases found in nature. In this context, halophilic archaea entail an excellent source of novel proteins that tolerate harsh conditions, as they live in environments with high salt concentration and temperature. In this work, a screening of haloarchaea, isolated from Odiel salterns in the southwest of Spain, was carried out to select a new strain with a high amylase activity. This microorganism was identified as *Haloarcula* sp. HS and showed amylase activities in both, the cellular and the extracellular extracts. Both amylase activities were poly-extremotolerant, as their optimal yields were achieved at 60 °C and 25% NaCl. Additionally, the study of the protein sequences from *Haloarcula* sp. HS allowed the identification of three different amylases, which conserved the typical structure of the alpha-amylase family. Finally, the applicability of the extracellular amylase to treat bakery wastes under high salinity conditions was demonstrated.

**Abstract:**

Alpha-amylases are a large family of α,1-4-endo-glycosyl hydrolases distributed in all kingdoms of life. The need for poly-extremotolerant amylases encouraged their search in extreme environments, where archaea become ideal candidates to provide new enzymes that are able to work in the harsh conditions demanded in many industrial applications. In this study, a collection of haloarchaea isolated from Odiel saltern ponds in the southwest of Spain was screened for their amylase activity. The strain that exhibited the highest activity was selected and identified as *Haloarcula* sp. HS. We demonstrated the existence in both, cellular and extracellular extracts of the new strain, of functional α-amylase activities, which showed to be moderately thermotolerant (optimum around 60 °C), extremely halotolerant (optimum over 25% NaCl), and calcium-dependent. The tryptic digestion followed by HPLC-MS/MS analysis of the partially purified cellular and extracellular extracts allowed to identify the sequence of three alpha-amylases, which despite sharing a low sequence identity, exhibited high three-dimensional structure homology, conserving the typical domains and most of the key consensus residues of α-amylases. Moreover, we proved the potential of the extracellular α-amylase from *Haloarcula* sp. HS to treat bakery wastes under high salinity conditions.

## 1. Introduction

Haloarchaea are the main representatives of extreme halophiles, which can thrive in media with salt concentrations ranging from 20 to 30% [1,2]. These singular microorganisms are characterized by the accumulation of large amounts of KCl in the cytoplasm, to maintain the osmotic balance with the medium, in contrast to moderate or facultative halophiles, which usually store compatible solutes for the same purpose [3]. Therefore, intra- and extracellular proteins from haloarchaea are specially adapted to work properly at high salt concentrations. These proteins possess exceptional features that make them distinguishable from non-halophilic proteins; they have a unique amino acid composition with acidic surfaces and low overall hydrophobicity, to prevent aggregation and, at the same time, retain flexibility in such high salinity [4]. Most haloarchaeal enzymes are considered poly-extremotolerant, as they work appropriately under more than one extreme condition, usually elevated temperatures, in addition to high salinity. For this reason, enzymes from these halophilic microorganisms can be of interest in many harsh industrial and biotechnological processes [5,6].

Amylases are a diverse group of hydrolase or transferase enzymes that degrade large alpha-linked polysaccharides, such as starch and related oligosaccharides, and are one of the most required enzymes in industrial operations. They stand for about 30% of the world enzyme market, and this value is expected to grow in the following years, due to the global increase in the demand for bakery and sugar-derived products, biofuels, detergents, breweries, animal feeds, pharmaceuticals, paper, and textiles [7,8]. At present, the best market for amylases is in the production of maltose and glucose syrups from corn, which are used as sweeteners for soft drinks [9,10]. They are also required for the enhancement of dough for baking, for clarification of fruit juices and beers, and in the pretreatment of animal feed to improve the digestibility of fiber [11]. In addition, amylases are widely used in textile and paper industries to remove the starch employed for the desizing process and the coating treatment, respectively. Moreover, several hydrolytic enzymes, including amylases, are usually added to detergents because they permit the use of milder conditions in laundry and automatic dishwashing, making them eco-friendlier. Other interesting fields of application of amylases are biomedicine and pharmacy, to treat digestive disorders or as reporter genes in molecular biology [12]. Furthermore, amylases are widely used for the conversion of starch-rich agronomic and food wastes into fermentable sugars, which are required as feedstocks for the production of fuels and chemicals with high demand and market value [13].

Amylases are ubiquitous ancient enzymes found in plants, animals, and microorganisms. Among them, bacteria of the genus *Bacillus* or fungi belonging to the *Aspergillus* genus are the most preferred source of amylases for large-scale production [14]. Hydrolytic amylases can be classified into two broad categories—endoamylases, which hydrolyze the interior of the starch molecule; and exo-amylases, which successively degrade starch from the non-reducing ends [9]. Most endoamylases belong to the α-amylase family (EC 3.2.1.1) and cleave internal α,1-4 glycosidic bonds between glucose units, producing oligosaccharides with varying lengths and α-limit dextrins. Additionally, α-amylases are typically divided into two groups, according to the hydrolysis products and the degree of starch hydrolysis; saccharifying α-amylases that produce free sugars, and liquefying α-amylases that break down the starch polymer without producing free sugars [5]. The mechanism of action and the catalytic properties of these amylases are well-known and can be correlated with their structural characteristics, as was detailed in several reviews [7,15,16].

The ability of haloarchaea to produce and excrete hydrolytic enzymes, including amylases to degrade extracellular polysaccharides, as many other microorganisms do, was previously described [6]. However, the application of archaeal amylases, which could be beneficial to many industrial operations that require extreme conditions, remains scarcely studied when compared to those from other microorganisms. Intracellular or cell-associated amylases from haloarchaea are particularly understudied, although they are an important haloarchaeal trait and can represent an interesting source of halotolerant enzymes.

The saltern ponds of the Odiel Marshlands are an interesting saline ecosystem, which harbors a rich diversity of prokaryotic and eukaryotic microorganisms. Our previous studies showed that at very high salinity (33%), the most abundant archaea species belong to the genera *Halorubrum* and *Haloquadratum* [17]. Metagenomic microbial profiling by high-throughput 16S rRNA sequencing revealed the existence of various strains that belonged to the *Haloarcula* genus. Although the abundance of these representatives was quite low, with less than 0.2% of the total sequenced reads, our data suggest that some of the haloarchaea of this group are able to produce bioactive compounds [18] and excrete hydrolytic haloenzymes, including proteases, amylases, or lipases [17].

In this study, a collection of haloarchaea isolated from the saltern ponds of the Odiel Marshlands was screened for their amylolytic activity, and the one that exhibited the highest activity was selected and identified on the basis of its 16S rRNA coding gene. The extracellular and cellular starch-degrading activities of the selected archaea were characterized, revealing different optimal parameters and modes of action. To get a further insight into the identity of these starch-degrading enzymes, the proteome composition of the partially purified cell-free supernatant and the cellular extracts was analyzed by tryptic digestion, followed by nano-liquid chromatography coupled to an electrospray ionization tandem mass spectrometry system. This study allowed the identification of three amylase sequences (two were exclusively cell-associated and one was also found in the extracellular medium) with high homology to amylases of other haloarchaeal species, and the typical alpha-amylase conserved regions. Furthermore, the potential applicability of the amylase enzymes of this new haloarchaea on the treatment of bakery waste was assessed and compared with a commercial amylase.

## 2. Materials and Methods

### 2.1. Screening and Selection of Amylase Producing Haloarchaea

The screening of amylase-producing haloarchaea was performed by detection of the extracellular amylase activity of the isolates on starch agar plates, with 20% NaCl. The plates were flooded with commercial Lugol’s iodine solution, 0.5% I_2_ and 1% KI (*w/v*) (Chem Lab, Zedelgem, Belgium), every three days, to check the formation of degradation halos around the colonies. The isolate that presented the highest ratio of halo zone with respect to colony diameter was chosen for further studies. Screenings were done in triplicates.

### 2.2. Identification of the Selected Microorganism

Genomic DNA of the isolated amylase-producing strain was purified using the GeneJET Genomic Purification kit (Thermo Fisher Scientific, Waltham, MA, USA), following the manufacturer’s instructions. The quantification and the purity assessment of the genomic DNA obtained was done on a Nanodrop Spectrophotometer ND-1000 (Thermo Fisher Scientific). The full length of the 16S rRNA encoding gene was amplified with the archaeal specific primers 21F (5′-TTCCGGTTGATCCTGCCGGA-3′) and 1492R (5′-GGTTACCTTGTTACGACTT-3′). Polymerase chain reactions (PCR) were performed in a total volume of 25 µL, using an Eppendorf thermo-cycler. The reaction mixture contained—1 µL of genomic DNA, 0.2 U REDTaq^®^DNA polymerase from Sigma Aldrich (St. Louis, MO, USA), and 2.5 µL of its specific 10× buffer that contained 10 pM of each primer, 0.2 mM dNTPs, and 2.5 mM MgCl_2_. The thermal profile was set to 0.5 min at 96 °C, 0.5 min at 55 °C, and 1 min at 72 °C for 30 cycles, followed by 10 min of final extension. The PCR products were analyzed by electrophoresis, on a 1% agarose gel to check their quality, and sent to Stabvida (Lisbon, Portugal) for Sanger sequencing. The 1.4 kb 16S rRNA gene sequences obtained were compared to those available at the GenBank and the European Molecular Biology Laboratory (EMBL) databases, using the Basic Local Alignment Search Tool (BLAST) at the National Center for Biotechnology Information (NCBI) [19].

### 2.3. Culture Conditions for Enzyme Production

All cultures were incubated at 37 °C with a shaking rate of 100 rpm, with either standard rich medium or minimal medium. The standard rich medium for archaea growth contained per liter—156 g NaCl, 13 g MgCl_2_·6H_2_O, 20 g MgSO_4_·7H_2_O, 1 g CaCl_2_·6H_2_O, 4 g KCl, 0.2 g NaHCO_3_, 0.5 g NaBr, and 5 g yeast extract, with a pH value of 7, measured before autoclaving. For the minimal medium, yeast extract was substituted for 1% (*w/v*) of ammonium acetate. The amylase activity was induced by the addition of starch (3 g L^−1^) to either the rich or the minimal medium.

### 2.4. Partial Purification of Cell-Associated and Extracellular Amylases

*Haloarcula* sp. HS cells were first cultured in the rich medium, containing yeast extract and starch. When the culture reached the end of the exponential phase (OD_580_ ≈ 3), cells were harvested through centrifugation, washed, and transferred to the minimal medium, where the biomass was cultivated until the extracellular starch was completely exhausted, about 3 days after the transference. Starch content was periodically measured every 24 h, by mixing 1 mL of culture medium with 5 µL of commercial Lugol’s iodine solution and by reading the absorbance at 580 nm. Then, the biomass was harvested by centrifugation for 20 min at 12,000 rpm and 4 °C. The supernatant was 100-fold concentrated by an ultrafiltration process in an Amicon^®^ system with a 10 kDa cut-off membrane and used as the source of the extracellular amylase. The specific amylase activity in the medium supernatant was 8 U mg^−1^ and it was increased to 350 U mg^−1^ in the concentrated supernatant, with a purification factor of 43.75. On its part, the cell pellet was disrupted by sonication in phosphate buffer (50 mM, 20% NaCl, pH 7) and centrifuged again to remove the cell debris and unbroken cells. The obtained cell extract was loaded onto a DEAE Sephacel^TM^ column equilibrated with the same phosphate buffer. The absorbed proteins were eluted using a linear gradient of NaCl from 0 to 500 mM and a final washing with NaCl 1 M, with a flow rate of 15 mL h^−1^. All fractions that presented amylase activity were collected and used as the cellular amylase source. In this case, the specific activity was increased from 20 U mg^−1^ in the crude cell extract to 120 U mg^−1^ in the partially purified preparation, with a purification factor of 6. Determination of the protein content in all the obtained extracts was performed according to the Bradford method [20], using bovine serum albumin (BSA) as standard.

### 2.5. Amylase Activity Assay

Unless otherwise indicated, the amylase activity was measured following the degradation of soluble starch by the standard iodine assay, based on the decrease of the absorbance at 580 nm of the iodine–starch complex produced by starch hydrolysis. The standard reaction mixture contained 50 µL of enzyme solution, 100 µL of 1% (*w/v*) potato starch solution in 20% NaCl, and 100 µL of phosphate buffer (50 mM, pH 7, 20% NaCl). The reaction mixture was incubated at 50 °C for 30 min, previously set as the best time to conserve the linearity of the activity. The reaction was stopped by cooling on ice and 100 µL were employed to reveal the remaining starch, by mixing 5 µL of four times diluted commercial Lugol’s iodine solution with the sample. Thereafter, 1 mL of distilled water was added to the sample before reading the absorbance at 580 nm. A standard curve was prepared with soluble starch. One unit of amylase activity was defined as the amount of enzyme degrading one microgram of starch per minute from soluble starch, under the assay conditions. To study the substrate specificity, potato starch was substituted by the indicated compounds (carboxymethyl cellulose, sucrose, and lactose) at a concentration of 1% (*w/v*), and incubated in the same conditions.

To analyze the starch hydrolysis products, aliquots were withdrawn from the incubation mixture at the initial reaction time and after 2 h of incubation at 50 °C. The reaction mixture contained 300 µL of the corresponding amylase extract and 600 µL of 1% (*w/v*) potato starch solution in 20% NaCl (*w/v*). The hydrolysis products were examined by a high-performance liquid chromatographic (HPLC) system (Merck-Hitachi LaChrom Elite), equipped with a refractive index detector (Merck-Hitachi L-2490) and an Aminex^®^ HPX-87H Column (Bio-Rad, Hercules, CA, USA), using an isocratic elution method with 5 mM H_2_SO_4_ at 50 °C, and a flow rate of 0.6 mL min^−1^. Glucose, maltose, and dextrin standards were obtained from Merck, Sigma-Aldrich (St. Louis, MO, USA).

### 2.6. Native Electrophoresis and Zymogram

The presence of amylase activity in the concentrated supernatant and the cell extract was revealed by in situ staining of a native PAGE containing 0.2% of soluble starch in the separating gel. A volume of 15 µL of the sample was mixed with 5 µL of loading dye and electrophoretically separated into two parallel gels of acrylamide, 10% supplemented with starch 0.2% (*w/v*) and run at 130 V. After electrophoresis, one of the gels was incubated in phosphate buffer (50 mM, pH 7, 20% NaCl) at 50 °C and 50 rpm for 1 h. Subsequently, the gel was stained with commercial Lugol´s reagent and the appearance of clear bands revealed the amylase activity. Meanwhile, the other gel was stained with 0.1% (*w/v*) Coomasie Brillant Blue R-250 in 45% (*v/v*) ethanol-10% (*v/v*) acetic acid, and faded with 25% (*v/v*) ethanol-10% (*v/v*) acetic acid. Molecular markers (NativeMark^TM^ Unstained Protein Ladder, Thermo Fisher Scientific, Waltham, MA, USA) were used as a reference for the molecular weight of proteins. Molecular mass estimation of the proteins was calculated by plotting the log (MW) as a function of Rf (migration distance of the protein/migration distance of the dye front).

### 2.7. Effect of NaCl, Temperature, pH, Metals, and Detergents on the Amylase Activities of the New Isolated Strain Haloarcula sp. HS

The effect of salt concentration was evaluated until a maximum of 32% NaCl with intervals of 4% salinity increase. The desired NaCl concentration was obtained by adding the required NaCl to the phosphate buffer (50 mM, pH 7) and to the 1% (*w/v*) starch solution. The influence of temperature on cell-associated and extracellular amylase activities was studied in phosphate buffer (50 mM, pH 7, 20% NaCl), over the range of 30–80 °C, with temperature increments of 10 °C. For pH studies, amylase activity was measured at 50 °C and 20% of salt, in the following buffers—50 mM acetate for pH 2 and 3; 50 mM MES for pH from 4 to 6; and 50 mM Tris-HCl for pH from 7 to 11. All the assays were done at least in triplicates, and the results were presented as a percentage of relative activity.

To test the influence of different metals on cell-associated and extracellular amylase activities MgSO_4_, CaCl_2_, CuCl_2_, FeCl_2_, FeCl_3_, or EDTA (ethylenediaminetetraacetic acid), were added to the reaction mixture, in a final concentration of 10 mM. Similarly, the effect of various surfactants were studied, including Tween20 (Polyoxyethylene (20) sorbitan monolaurate), Tween80 (Polyoxyethylene (80) sorbitan monooleate), Triton-X100 (2-[4-(2, 4, 4-trimethylpentan-2-yl) phenoxy] ethanol), CHAPS (3-[(3-Cholamidopropyl) dimethylammonio]-1-propanesulfonate), SB-12 (N-Dodecyl-N,N-dimethylammonio-3-propane sulfonate), and SDS (sodium dodecyl sulfate), in a final concentration of 0.5% (*w/v*). Amylase residual activity was measured as previously detailed for the standard assay and expressed as a percentage, with respect to a control sample incubated in the absence of additives. All the determinations were conducted in triplicates.

### 2.8. Proteomic Analysis

For the proteomic analysis, the concentrated supernatant and the partially purified cell extract fractions with starch degrading activity were dialyzed for 48 h, against a solution of 1% NaHCO_3_ and 0.01% EDTA in milli-Q water, to eliminate excess salt. The proteins were first precipitated with TCA/acetone and resuspended in ammonium bicarbonate-trifluoroethanol (50%). After that, the proteins were treated with dithiothreitol, 10 mM, and methyl ethanethiosulfonate, 10 mM. Prior to trypsin digestion, the samples were diluted with ammonium bicarbonate 25 mM, until the concentration of trifluoroethanol was under 5%. The digestion with trypsin was done overnight at 37 °C. Subsequently, the samples were analyzed by LC-MS/MS in a triple quadrupole-TOF system (5600 plus, ABSciex, Vaughan, ON, Canada), equipped with a nano-electrospray ion source, coupled to a nano-HPLC (Eksigent, Vaughan, ON, Canada). The Analyst TF 1.7 software was used for equipment controlling and data acquisition. Peptide mass tolerance was set to 25 ppm and 0.05 Da, for fragment masses, and only 1 or 2 missed cleavages were allowed. The peptide and protein identifications were performed using the Protein Pilot software (version 5.0.1, SCIEX, Vaughan, ON, Canada), with the Paragon algorithm. The search was conducted against the Uniprotproteome_Haloarcula_hispanica database 11_24_2020. The false discovery rate (FDR) was set to 0.01 for both peptides and proteins. Protein comparison was performed with the Basic Local Alignment Search Tool for proteins (BLASTp) of the NCBI (National Center for Biotechnology Information). The obtained sequences were analyzed using the CLC Workbench software (version 8, Qiagen, Hilden, Germany).

### 2.9. Identification of Amylase Coding Genes Based on Protein Sequences

With the aim of completing the full protein sequences of the amylases identified, the sequences of their encoding genes were amplified by PCR, using sets of primers specifically designed on the basis of the sequences of peptides obtained in the proteomic analysis. Concretely, six pairs of primers were employed to cover almost the full length of the DNA sequences of the three amylases found, obtaining two overlapping sequences for each amylase gene (Table 1).

Polymerase chain reactions were performed in a total volume of 25 µL, using an Eppendorf thermo-cycler. The reaction mixtures contained—1 µL of genomic DNA, 0.2 U REDTaq^®^DNA polymerase from Sigma Aldrich (St. Louis, MO, USA), and 2.5 µL of its specific 10× buffer that contained 10 pM of each primer, 0.2 mM dNTPs, and 2.5 mM MgCl_2_. The thermal profile was set to 0.5 min at 96 °C, 0.5 min at 62 °C, and 1 min at 72 °C for 30 cycles, followed by 10 min of final extension. The PCR products were analyzed by electrophoresis on a 1% agarose gel and sent to Stabvida (Lisbon, Portugal) for Sanger sequencing. The sequences obtained were translated to protein and both, DNA and protein sequences, were compared to those available at National Center for Biotechnology Information (NCBI) databases, using the Basic Local Alignment Search Tool (BLAST). Finally, different alignments were conducted in the CLC Workbench software (version 8, Qiagen), the predicted structural models were built using the Phyre2 [21] and NetSurfP [22] online web servers, and three-dimensional (3D) molecular graphics were analyzed in the UCSF Chimera version 1.15 [23] (University of California, Oakland, CA, USA). Physicochemical characteristics of the proteins were obtained using the ProtParam tool (ExPASy) [24]. 

### 2.10. Starch Hydrolysis from Bakery Waste

Bread from bakery waste was chosen for the present experiment. Bread crumbs were dried on a stove at 70 °C and milled in a porcelain mortar to obtain a fine powder. Starch was recovered by mashing dried crumbs in distilled water, in saltwater at 20% NaCl (*w/v*), and in saturated saline solution (33% NaCl). The ability of the extracellular amylase of *Haloarcula* sp. HS to degrade the starch from bread was comparatively tested against a commercial α-amylase (Megazyme cat. no. E-BSTAA). Starch and enzyme solutions were mixed in a proportion of 1:1 (*v/v*) in a final volume of 1 mL. The hydrolysis of the starch was performed for 15 min at 60 °C in 50 mM acetate buffer pH 5. The amount of remaining starch was measured by the iodine-starch method. A control, containing starch recovered from bread without the enzyme solution, was incubated in the same conditions. All assays were conducted in triplicates.

## 3. Results

### 3.1. Selection of Amylase-Producing Haloarchaea Isolated from Odiel Salterns Ponds

**Figure 1:**
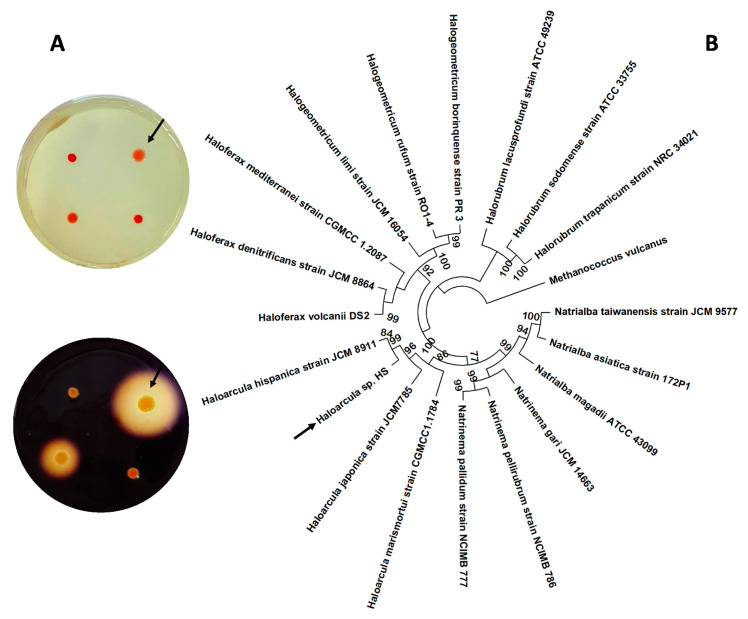
(**A**) In vitro selective screening for amylase-releasing haloarchaea. Semi-quantitative estimation of amylase activity from four haloarchaeal strains isolated from the Odiel Marshlands, grown on starch agar plates (**top**) and revealed with Lugol´s iodine solution (**down**), as detailed in the Material and Methods section. (**B**) Molecular phylogenetic analysis by the maximum likelihood method. The tree represents a comparison among the complete 16S rRNA coding gene sequences, including a series of reference haloarchaeal species and the new isolated strain, *Haloarcula* sp. HS. Multiple alignments were generated by MUSCLE (MUltiple Sequence Comparison by Log-Expectation) and the tree was constructed with MEGA X. The numbers at the nodes indicate the bootstrap values calculated for 1000 replicates. Arrows point to the new strain *Haloarcula* sp. HS.

An in vitro screening was carried out to select the best amylase-producing strain, among a collection of archaea previously isolated from the saltern ponds of the Odiel Marshlands (SW, Spain) with a salinity of 33%. Amylase activity of each isolate was screened for 9 days on starch-agar plates, as detailed in Material and Methods (Figure 1A). Eight colonies showed a considerable amylase activity, and that with the largest halo was selected and identified, by amplification and sequencing of its 16S rRNA full-length coding gene (Supplementary Material, Figure S1), followed by the comparison of the obtained sequence with the NCBI database using the BLASTn tool. The results showed that the selected strain was closely related to the *Haloarcula* genus, showing 98% homology with different species of this taxonomic group. Therefore, the new strain isolated was named *Haloarcula* sp. HS.

Molecular phylogenetic analysis was performed using the Molecular Evolutionary Genetics Analysis (MEGA X) [25], on a series of reference haloarchaeal species and on the new isolated strain, *Haloarcula* sp. HS (Figure 1B). The hyperthermophilic archaea *Methanococcus vulcanus* was used as an outgroup and the bootstrap was set at 1000 replicates. The 16S rRNA encoding sequence of the isolate clustered with the corresponding genes of the representatives of the *Haloarcula* genus, especially close to the species *Haloarcula hispanica.*

### 3.2. Optimization of a Two-Stage Culture Strategy to Induce the Production of Amylase

In the studied archaea, significant levels of amylase activity were only found when the biomass was grown under inductive conditions in the presence of starch. The culture conditions that induced the production of amylases are widely studied for bacteria and hyperthermophilic archaea, as reviewed by Mehta and Satyanarayana [5], but more limited information exists on the production of amylase in haloarchaea [26,27]. Most authors agree that amylase production is growth-associated and is strongly induced by starch.

**Figure 2:**
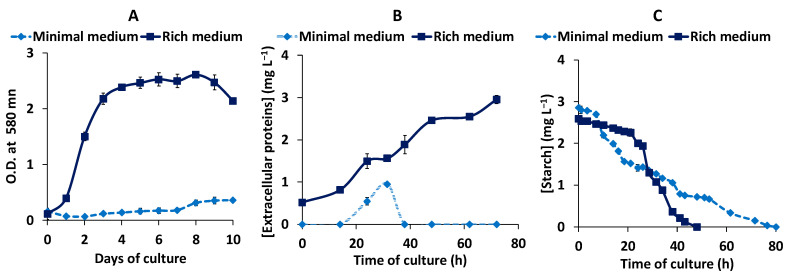
Time course evolution of *Haloarcula* sp. HS cultures in rich and minimal media. Optical density (**A**), secretion of proteins (**B**), and starch hydrolysis (**C**) were measured during the time of culture in rich (■) and minimal (♦) broths. All data are expressed as the mean ± SD of at least triplicate experiments.

To establish the best culture conditions for the production and excretion of amylase by *Haloarcula* sp. HS, the haloarchaea was cultured in a (i) rich medium, which contained yeast extract and a (ii) minimal medium, in which the yeast extract was substituted by ammonium acetate. In both cases, starch (3 g L^−1^) was added to the culture medium, as detailed in Materials and Methods. The optical density of the cultures, protein secretion into the media, and hydrolysis of extracellular starch were followed in both, rich and minimal medium cultures. As shown in Figure 2, cell growth and protein secretion were higher when the microorganism was grown in the rich media, which contained yeast extract. In this medium, the studied archaea reached the stationary phase of growth in about 4 days and excreted 3 mg L^−1^ of proteins into the culture medium. The archaea cultured in the minimal medium exhibited very slow growth and excreted much fewer proteins to the culture medium, about 1 mg L^−1^, after 30 h of culture. However, starch degradation activity was much higher for the archaea cultured in the minimal medium, which despite a much lower biomass, showed an initial starch degradation rate 5.5 times higher than that of the rich medium. Although starch was completely hydrolyzed in both media, there was a 24 h lag phase before starch degradation started in the medium with yeast extract, probably due to the presence of more easily assimilable carbon sources in this medium.

**Figure 3:**
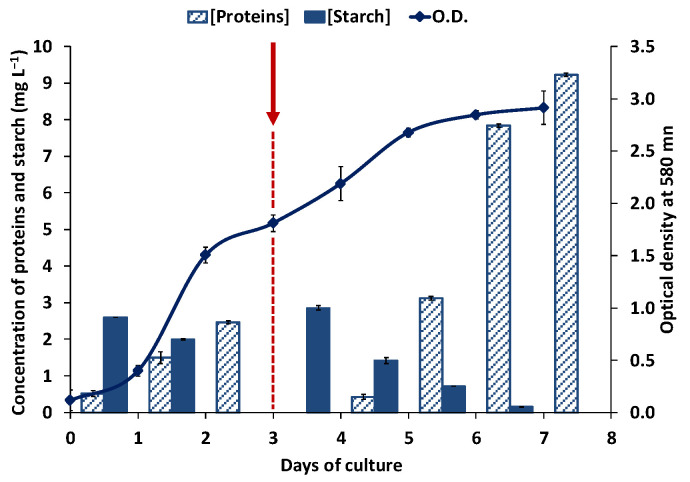
Time course evolution of *Haloarcula* sp. HS in the two-step culture. Optical density, secreted proteins, and starch concentration were measured along the full cultivation time. The red arrow indicates the moment of transference of the cells from the rich to the fresh minimum medium. All data are expressed as the mean ± SD of at least triplicate experiments.

For this reason, a two-step culture was set up to get both, a high biomass and amylase productivity. Cells were first grown in a rich medium in order to obtain a large amount of biomass, and when the culture reached the end of the exponential phase of growth, at the third day of culture, the cells were transferred to the fresh minimal medium to induce the production of amylase, and was cultured for another 4 days. Through this two-step approach, high starch consumption activity and a high protein excretion were achieved, reaching an extracellular protein concentration of 10 mg L^−1^ and undetected levels of extracellular starch, on the 7th day of culture (Figure 3).

### 3.3. Extracellular and Cell-Associated Amylase Activities in Haloarcula sp. HS

**Figure 4:**
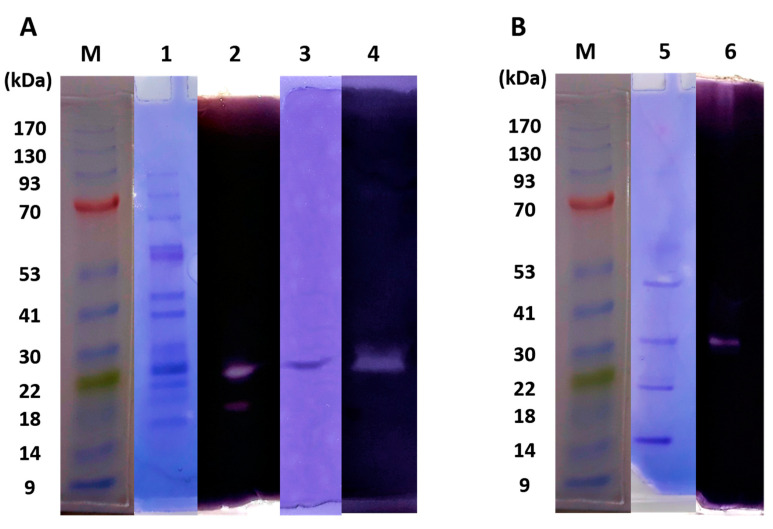
Native-PAGE and zymogram of cell-associated (**A**) and extracellular (**B**) proteins obtained from *Haloarcula* sp. HS. Lanes 1, 3, and 5—samples on native-PAGE followed by Coomassie Blue staining. Lanes 2, 4, and 6—samples on native PAGE followed by Lugol´s solution staining. Lane M—molecular mass marker in kDa, lanes 1 and 2—crude extract, lanes 3 and 4—partially purified crude extract, and lanes 5 and 6—concentrated supernatant. The whole gels for each staining are available in Supplementary Material, Figure S2.

To identify the enzyme responsible for the amylase activity and characterize its properties, the haloarchaeal strain *Haloarcula* sp. HS was grown in a two-stage culture with starch (3 g L^−1^), as previously described (Figure 3). Cell-associated proteins and the concentrated extracellular proteins excreted into the cultured medium were electrophoretically separated in a polyacrylamide gel containing starch. After electrophoresis, the gel was split lengthwise with a razor blade. One half was stained with Coomassie blue and the other with Lugol’s iodine solution to detect both proteins and amylase activity, respectively. The zymogram analysis proved the presence of amylase activity in both samples, the culture medium, and the crude extract. A unique band with amylase activity was observed in the culture medium, after a 100-fold concentration step through ultrafiltration with a 10 kDa cut-off membrane, as indicated in Material and Methods. However, in the crude extract, two bands with starch hydrolyzing activity were observed, indicating the presence of several cell-associated enzymes with amylase activity (Figure 4 and Figure S2). The crude extract was partially purified through ion-exchange chromatography in DEAE Sephacel^TM^, as indicated in the Materials and Methods section. All fractions with amylase activity were pooled and used as the source of cellular amylase. The electrophoretic analysis of the purified extracts showed a unique band with amylase activity (Figure 4A). The size of the observed bands was between 20 and 27 kDa, however, the protein mobility was strongly affected by the starch added to the polyacrylamide gel and these apparent sizes observed were not representative.

### 3.4. Characterization of Extracellular and Cell-Associated Amylase Activities

A series of in vitro experiments were carried out with the extracellular and cellular amylase-enriched extracts to characterize the hydrolysis products, the substrate specificity, and the optimal kinetic parameters of the amylase activity of these extracts. Both, extracellular and cellular, enzymatic preparations were incubated with 1 mg of starch in the standard conditions, described in Material and Methods, excepting that the incubation time was fixed at 2 h. The products of starch hydrolysis were identified by HPLC and an IR detector, as detailed in Materials and Methods. A parallel reaction with a commercial α-amylase purchased from Megazyme (cat. no. E-BSTAA) was done in the same conditions for comparison. The results (Table 2) revealed that starch degradation was almost complete in all cases, being especially efficient in the case of the extracellular amylase extract, with a remaining starch of only 1.7%. The main product obtained with the three enzymatic sources was maltose, which represents between 73.8% and 86.1% of the total carbohydrate content in the reaction mixtures. In addition, the enzymatic preparation from the cellular extract was also able to catalyze the liberation of glucose (6.6%). On the other hand, the extracellular extract and the commercial reference amylase catalyzed the liberation of dextrins, which supposed 18.5% and 20.8% of the total carbohydrate content in the reaction mixture, respectively, in addition to maltose. No glucose was found as the end product in these reactions (Table 2).

With respect to the substrate specificity, neither extracellular nor cellular *Haloarcula* sp. HS extracts were able to hydrolyze other glucose polysaccharides, such as carboxymethyl cellulose, or disaccharides, such as sucrose or lactose, which not contain alpha-1,4-linked glucose.

**Figure 5:**
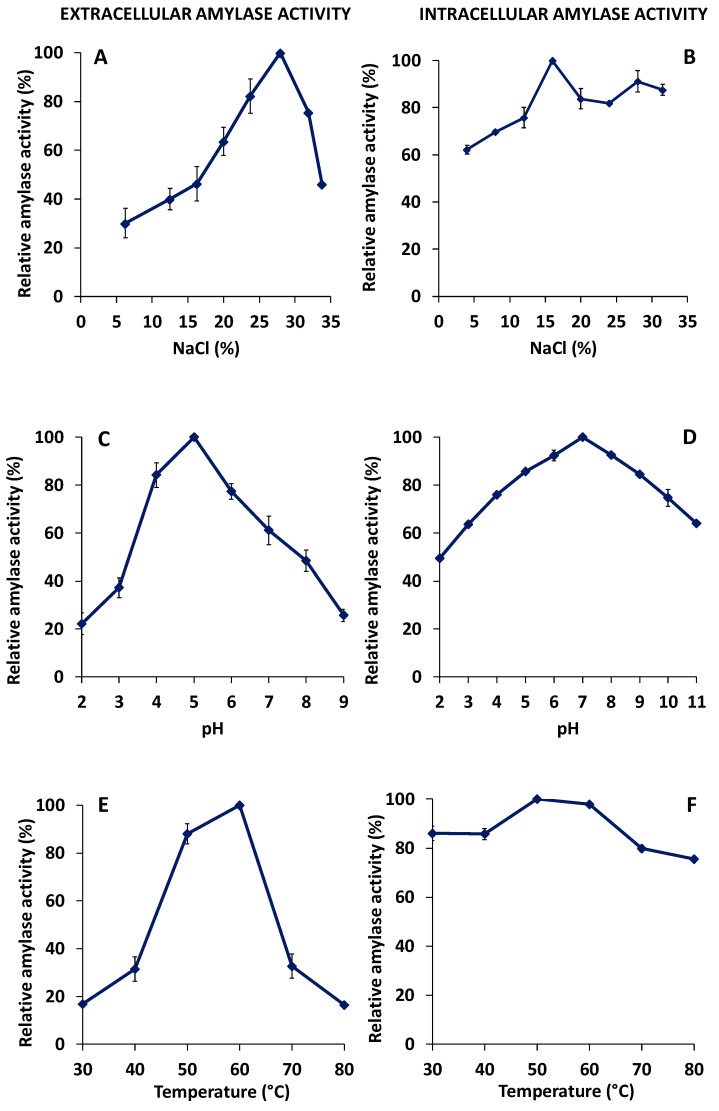
Effect of salt, pH, and temperature on amylase activities. Relative amylase activities in different salt concentrations (%, *w/v*), pH values, and temperatures are shown for the extracellular (**A**,**C**,**E**) and cellular (**B**,**D**,**F**) extracts. Relative activity was defined as the percentage of maximum activity for each case. The 100% activity corresponded to 70 ± 6.4 U mL^−1^ (350 U mg^−1^) for the extracellular amylase extract and 60 ± 5.6 U mL^−1^ (120 U mg^−1^) for the mix of cell-associated amylases. Mean and standard deviations are shown.

The most characteristic feature of halophilic enzymes is their ability to operate under very high salinities. As it is shown in Figure 5, the extracellular enzymatic preparation presented the optimal activity at 28% salt and retained less than half of its activity when the salt content was under 20% (Figure 5A), showing that it is more salt-dependent than the cellular one, which instead showed the maximum activity at 16% salinity and retained more than 60% of its activity at all salinities studied (Figure 5B). 

The influence of pH in both amylase activities was studied in different buffers, as detailed in Materials and Methods. The optimal activity was found at pH 5 for the extracellular amylase, and at pH 7 for the cellular amylase-enriched extract, as shown in Figure 5C,D, respectively. It should be noted that the cell-associated activity was stable under a wide range of pH values, retaining more than 50% activity at pH values between 2 and 11, and more than 80% activity at pH values comprising pH 5 to 9. Contrarily, the extracellular amylase activity appeared to be more susceptible to extreme pH, losing more than 50% of its activity both at low and high pH values.

The effect of the temperature on the amylase activities showed, once again, that the extracellular amylase activity had a higher dependence on the physicochemical parameters of the assay than the cell-associated one. The extracellular amylase activity showed an optimal temperature of 60 °C, losing more than 65% of its activity below 50 °C or above 60 °C (Figure 5E). The cellular amylase-enriched extract, on the other hand, conserved a high activity over a wide range of temperatures, retaining more than 75% of activity from 30 to 80 °C (Figure 5F). This weak temperature dependence was due to the fact that a mix of three different cell-associated enzymes could contribute to the amylase activity, as later shown by the proteomic analysis of the cellular enzymatic preparation.

### 3.5. Effects of Metals and Surfactants on the Amylase Enzymatic Activities

**Figure 6:**
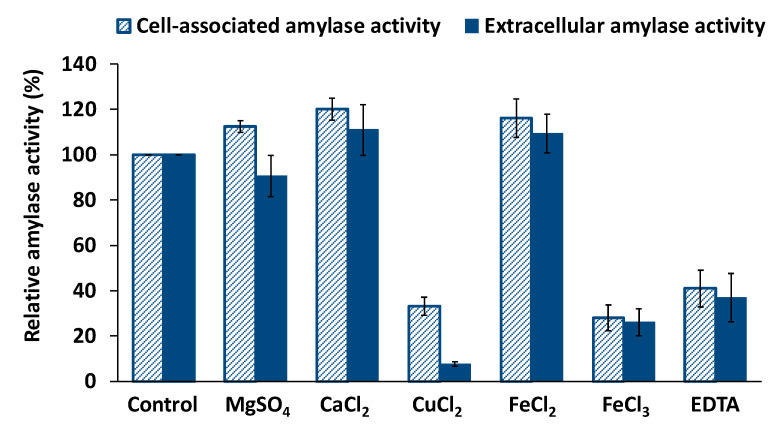
Effect of metal ions on the amylase activities. Extracellular and cell-associated relative amylase activities under the presence of different metal ions and EDTA (10 mM) are represented. Relative activity was defined as the percentage of maximal activity with respect to control, with no additives. The control activity was 70 ± 6.4 U mL^−1^ (350 U mg^−1^) for the extracellular amylase and 60 ± 5.6 U mL^−1^ (120 U mg^−1^) for the cellular amylase. Mean and standard deviations are shown.

Many microbial α-amylases are reported to be calcium-dependent metalloenzymes [9]. Therefore, the effect of EDTA, Ca^2+^, and other metallic ions including Mg^2+^, Cu^2+^, Fe^2+^, and, Fe^3+^ was tested. The results revealed that the addition of Ca^2+^, Fe^2+^, or Mg^2+^ causes a slight increase in the amylolytic activity of both extracellular and cellular enzymatic extracts (Figure 6). The possible existence of divalent metals in the partially purified enzymatic preparations makes it difficult to obtain accurate conclusions on the effects of these metals on the amylase activities of *Haloarcula* sp. HS. However, the strong inhibition observed in the presence of the metal chelating agent EDTA confirmed the divalent cation dependence of both, extracellular and cell-associated amylase activities, which decreased to 39% and 33%, respectively, in the presence of EDTA.

On the other hand, the presence of Cu^2+^ and Fe^3+^ in the reaction mixture, at a concentration of 10 mM, caused a drastic reduction in both, extracellular and cell-associated amylase activities (Figure 6). The cellular extract only retained 28% of its amylase activity in Fe^3+^; and similarly, the extracellular amylase conserved 26% of its activity. Under the presence of the cupric ion, the activity of the extracellular amylase dropped to 8%, while the activity of the cell-associated amylase decreased to 33%. This fact suggests the involvement of tiol/carboxyl groups, typically inhibited by Cu^2+^, in the function of the enzymes [28].

The in vitro effect of anionic (SDS), cationic (SB-12), zwitterionic (CHAPS), and no ionic (Tween 20, Tween 80, and Triton X-100) detergents on the amylase activities were assayed. The obtained results revealed that both activities were very stable in different detergents, retaining more than 80% activity in all, with the exception of SDS, which caused a decrease by almost half in the amylase activities of both, extracellular and cellular amylase enriched extracts.

### 3.6. Identification of Amylases in Cellular and Extracellular Concentrated Extracts of Haloarcula sp. HS by a Proteomic Approach

A proteomic study of both, extracellular and cellular partially purified extracts with amylase activity, was carried out to identify the sequence of the proteins responsible for these starch-degrading activities in *Haloarcula* sp. HS. Both samples were submitted to tryptic digestion and analyzed by LC-MS/MS in a triple quadrupole-TOF system, as described in Materials and Methods.

The results revealed that the main extracellular protein secreted to the culture media was α-amylase. The rest of the proteins identified in the extracellular fraction were membrane-ligated proteins, probably from broken cell remains. This extracellular amylase (AMY_HS1) was identified by 42 unique peptides, presenting 73.56% identity and 60% query cover with the α-amylase of *Haloarcula hispanica* N601 (UniProt: V5TMJ3_HALHI). For its part, in the cellular fraction, three different amylases were found. One of them corresponded to the same α-amylase (UniProt: V5TMJ3_HALHI) detected in the extracellular fraction, which in this case was identified according to 13 unique peptides and showed 81.48% identity and 36% query cover. The other two proteins, denoted as AMY_HS2 and AMY_HS3, showed high homology with two different α-amylases of *Haloarcula hispanica* N601 (UniProt codes: V5TRA6_HALHI and V5TQD3_HALHI, respectively), both determined according to 15 unique peptides. AMY_HS2 presented 45.25% identity and 65% query cover with V5TRA6_HALHI, while AMY_HS3 showed 75.76% identity and 31% query cover with V5TQD3_HALHI.

Due to the low percentage of protein covering achieved, the sequences of amylase coding genes were amplified by PCR, as detailed in Material and Methods, with primers designed to target the peptide sequences identified by proteomics for each enzyme. Through this approach, practically the full length of each protein sequence was completed.

**Figure 7:**
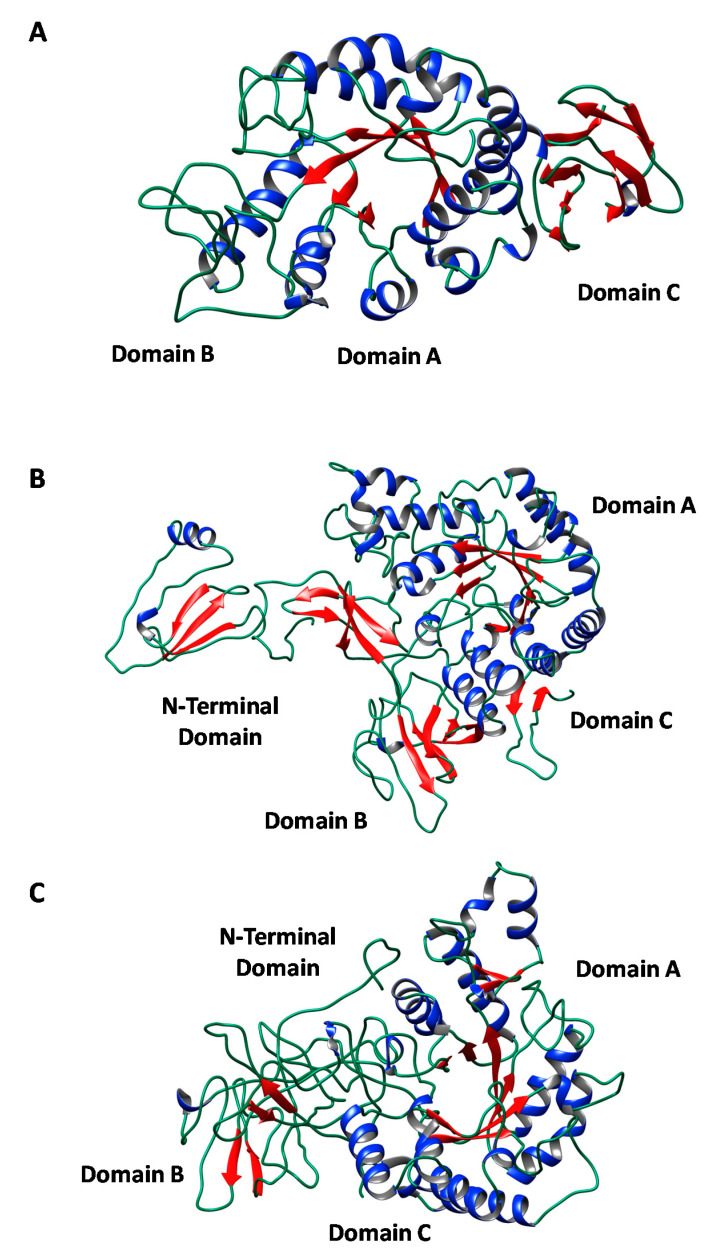
Three-dimensional predicted structures of the three amylase sequences identified in *Haloarcula* sp. HS; (**A**) mature extracellular amylase, AMY_HS1; (**B**) cellular amylase, AMY_HS2; (**C**) cellular amylase, AMY_HS3. Phyre2 software and the Chimera program were employed for 3D structure visualization. Helixes are represented by blue ribbons and strands by red arrows.

The alignment of the three obtained protein sequences revealed that the extracellular amylase only presented a 17% identity with the cell-associated amylases, which in turn showed a 38% identity between them. Nonetheless, as it is shown in their predicted three-dimensional (Figure 7) and secondary structures (Supplementary Material, Figure S3), the three amylase sequences from *Haloarcula* sp. HS conserve the typical structural domains of the GH-13 family, according to the Carbohydrate-Active enZYmes (CAZY) database; including the catalytic (β/α)_8_-barrel (TIM-barrel) located in the domain A, a small domain B located in the loop between the β_3_-strand and the α_3_-helix of the barrel, and the domain C showing an antiparallel β-sandwich structure in the C-terminal end of the protein. In addition to this typical conformation, the two cellular amylases show an N-terminal domain, which was reported in some maltogenic amylases and seems to be involved in increasing the binding of the enzyme to raw starch. This N domain forms a large groove, the N–C groove, which might be responsible for thermostabilization via oligomerization and substrate affinity modifications in some microbial maltogenic amylases [29]. For its part, the extracellular protein, AMY_HS1, presents the conserved TAT (Twin-Arginine-Translocation) motif and its corresponding processing site (Supplementary Material, Figure S4).

Regarding the physicochemical characteristics, the three alpha-amylases of *Haloarcula* sp. HS had a low isoelectric point, negative net charge, and low hydrophobicity (Table 3) as other haloarchaeal enzymes.

**Figure 8:**
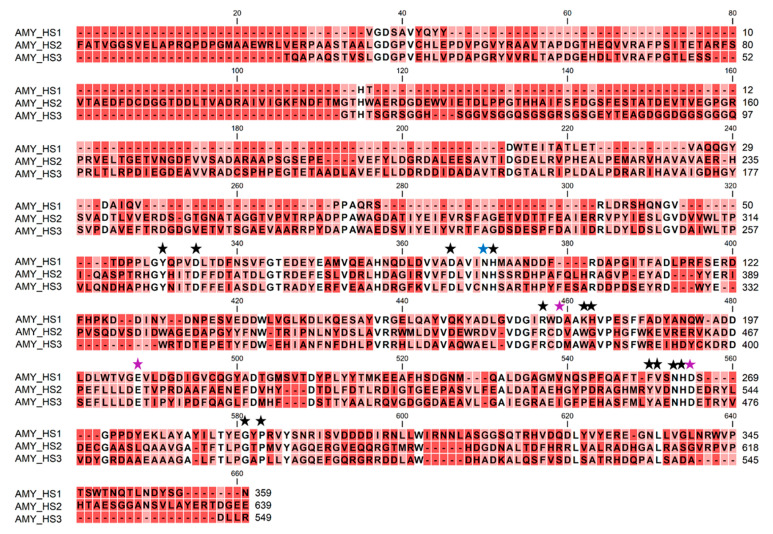
Alignment of the three amylase sequences from *Haloarcula* sp. HS. The mature extracellular protein is named AMY_HS1, while the cell-associated amylases are denoted as AMY_HS2 and AMY_HS3. Purple stars highlight the catalytic triad (Asp-Glu-Asp), blue star denotes the canonical calcium-binding site and the black stars point other essential residues for enzyme structure. Identical residues in the three sequences are shaded in white, residues that coincide in two of the sequences or do not coincide at all are shaded in pink and red, respectively.

Moreover, although the three protein sequences had a low percentage of sequence homology, they conserved the catalytic triad of aspartate, glutamate, and aspartate in the active site, along with other conserved residues that were described to be indispensable for the structure of the enzyme [30]. These conserved amino acids are shown in Figure 8. One of the first consensus amino acids found is aspartic acid, which is essential for active site integrity. This aspartic residue is in the position Asp92 for the mature AMY_HS1 protein after TAT processing, and occupies the position 359 and 303 in AMY_HS2 and AMY_HS3, respectively (Asp92/359/303). Following the same notation, the rest of the conserved amino acids are distributed as follows—Asn96/363/307, which coordinates the conserved calcium ion between the A and B domains [31]; and His93/360/304, which stabilizes the interaction between the C-terminal of β3 and the rest of TIM barrel through hydrogen bonding to Asn61/328/272 and the backbone oxygen of Tyr57/324/268. The first catalytic residue is Asp177/446/379, located in β4, which is preceded by Arg175/444/377, both of which are indispensable amino acids for the catalytic activity. Lys and His are usually present in this region in the position Asp+3 and +4, binding the reducing end of the glucose chain in the substrate-binding cleft [32], however, these residues were only found in the extracellular amylase. The second catalytic residue is the proton donor Glu205/475/408, which lies in the fifth L-strand of the TIM-barrel. The following conserved residues protect the active site from the solvent and contain the last catalytic amino acid Asp268/539/471, postulated to be involved in substrate binding, substrate distortion, and in elevating the pKa of Glu205/475/408. This residue is usually accompanied in α-amylases by His, Asn, Val, and Phe in the positions −1, −2, −4, and −5, respectively, as it occurs in AMY_HS1, while in AMY_HS2 and AMY_HS3, Phe is substituted by Tyr, and Val is changed by Ala in AMY_HS3. Finally, the other two conserved residues were found in Gly287/564/496, followed by Pro289/566/498 [33,34,35,36].

Finally, a multispecies study was carried out to compare the degree of conservation among the amylases reported and those from different haloarchaeal members. The protein sequence of the extracellular amylase (AMY_HS1) was aligned and compared to various extracellular amylase sequences available in the NCBI database, selecting different representatives of the order *Halobacteriales* (Supplementary Material, Figure S4). Among these amylase sequences, different percentages of identity were found with the extracellular amylases from *Haloarcula hispanica* N601 (90%), *Halomicroarcula salina* (73%), *Halapricum salinum* (57%), and *Haloterrigena turkmenika* (40%). Likewise, the two cell-associated amylases (AMY_HS2 and AMY_HS3) from *Haloarcula* sp. HS showed the following percentages that identity with the amylase sequences from *Haloarcula hispanica* N601 (90 and 80%, respectively), *Halomicroarcula salina* (70 and 65%, respectively), *Haloferax mediterranei* (59, and 50%, respectively), *Halogeometricum rufum* (57% with AMY_HS2), and *Halogeometricum limi* (49% with AMY_HS3) (Supplementary Material, Figures S5 and S6). In all alignments, it could be appreciated that the catalytic regions were conserved among the different haloarchaeal species (Supplementary Material, Figures S4–S6).

### 3.7. Hydrolysis of Bakery Waste

Bakery residues, including dough, flour dust, burnt or rejected bread, and biscuits, can be exploited for the production of fermentable sugars. Among them, bread was chosen as the substrate for this assay, as it is one of the most abundant food waste products worldwide. In addition, many of the discarded by-products during the bread manufacturing process are fundamentally constituted by starch, such as substandard bread and the bread crust removed to make special types of sandwich bread [37].

The extracellular amylase activity was selected for this assay, as it works better in high NaCl concentrations than the cell-associated amylase. This was compared to a commercial thermostable α-amylase. Sodium chloride was found to have a complex effect on the gelatinization and rheological properties of starch. Some studies pointed out that the enthalpy for the gelatinization process decreased at high salt concentrations, an effect of great importance for the production and properties of several cereal-based products, as well as for the manufacture of modified starches [38].

The results proved that a high percentage of the initial starch (75–85%) was hydrolyzed by both enzymes, with maximum activities above 100 U mL^−1^. However, the concentration of salt was determined in their maximum activities. The extracellular amylase hydrolyzed around 75% of the starch under 20% salt or even under salt saturation, with maximum activities of 107 and 105 U mL^−1^, respectively; while when no salt was added, the degradation rate decreased to 22%, with an activity of 24.7 U mL^−1^. Conversely, the commercial α-amylase presented the highest activity (101.8 U mL^−1^) when no salt was added to the mixture, hydrolyzing 85% of the starch; and its activity dropped drastically to 6–7 U mL^−1^, under elevated salt concentration, degrading only 5–6% of the starch.

The obtained hydrolysate was very rich in simple sugars that could now be used for the bioproduction of many high-value molecules like glycerol, hydrogen, ethanol, or lactic acid, among others. These molecules are required for different purposes, including renewable energy sources, fuel additives, and food preservers [13]. To our knowledge, this study supposes the first attempt to use a halophilic amylase to degrade starch from bread into simple sugars. The applicability of the amylase from a haloarchaea was tested using starch from agricultural waste [39], however, the starch content of this residue was considerably lower, and also the starch extraction method was costlier.

## 4. Discussion

Alpha-amylases are a large family of endo-glycosyl hydrolases that cleave the internal α,1-4 glycosidic bonds between the glucose units in polysaccharides, such as amylose and amylopectin. They are common in all kingdoms of life and their general properties, three-dimensional structures, and mechanisms of action are extensively reviewed [40,41,42,43].

Alpha-amylases are particularly spread among microbial species, such as bacteria and yeasts. These amylases are often extracellular enzymes that allow microorganisms to use environmental polysaccharides for their nutrition. Thermostable α-amylases were produced and commercially exploited from yeast and bacterial species, such as *Bacillus subtilis*, *B. licheniformis*, or *B. amyloliquefacines* [14]. Some archaea, including halophilic archaea, were found to produce halotolerant α-amylases, which in addition were highly or moderately thermotolerant. The extracellular α-amylases of *Haloarcula* sp. S-1 [44], *N. amylolyticus* [45], *H. mediterranei* [26], *H. xinjiangense* [46], *Haloferax* sp. HA10 [47], *H.turkmenica* [39], or *Halococcus* GUVSC8 [48] are some examples. Although much less studied, the intracellular α-amylases of some haloarchaea, such as *Haloarcula japonica* [49] were also characterized.

The new strain isolated from the Odiel Marshlands and selected by its high ability to degrade starch in iodine–starch agar plate assays was foumd to be closely related to the genus *Haloarcula*, as shown in the phylogenetic tree (Figure 1). The high homology of its 16S rRNA with that of *Haloarcula hispanica* (98%) *or Haloarcula japonica* (97%) confirmed it. We found that the new strain exhibited a high amylolytic activity when cultured in the presence of starch; this activity was higher in a minimal medium with ammonium acetate (Figure 2).

In agreement with our observations, several reports indicate that amylase production in haloarchaea is induced by starch, as described for *Halorubrum* [46], *Haloferax* [47], *Haloarcula* [50], and some halophilic bacteria [51]. Other culture conditions and nutrients that were reported to influence the induction of alpha-amylases are nitrogen, metal ions, or phosphate [5]. Pérez-Pomares et al. [26], for example, reported low amylase excretion when using a minimal medium containing ammonium acetate as carbon and nitrogen source, plus starch in *Haloferax mediterranei.*

Our observations indicate that the conditions that induce the amylase activity are not the best for growth (Figure 2). Therefore, a two-step culture was set up, in which a large amount of biomass was obtained in a rich medium, followed by transfer to a minimal medium with starch, to induce the production of amylase activity (Figure 3). This two-stage method allowed the improvement of amylase production, and at the same time facilitated the recovery of the extracellular enzyme. Since the minimal medium had no yeast extract, there are no foreign proteins that could interfere with the amylase secreted into the culture medium.

Partially purified extracellular and cellular-amylase-enriched extracts were obtained from *Haloarcula* sp. HS, through ultrafiltration and anion exchange chromatography, respectively, and were electrophoretically separated in native conditions. In situ staining of the obtained acrylamide gel allowed the identification of bands with starch degrading ability, one band in the extracellular enzymatic preparation, and two bands in the cellular extract, with apparent relative molecular masses between 21.6 and 30.4 kDa (Figure 4). The zymogram indicates that the new isolated strain presents amylase activities in both, the supernatant and the crude cell extract, suggesting that there is more than one cell-associated amylase that does not coincide with the extracellular one. The proteomic analysis of the extracts and the subsequent amplification of the whole gene sequences that encode for these amylases allowed us to confirm this hypothesis (Figure 8), and also indicated that the real masses of the amylases were much higher than the apparent molecular masses shown in the electrophoresis gel. These differences could be due to the fact that the electrophoresis was carried out in native conditions and in the presence of starch, which could modify their electrophoretic mobilities, besides the fact that halophilic proteins usually show altered electrophoretic properties [52].

The apparent molecular masses reported for the amylases of other haloarchaea are slightly higher than the molecular weight observed for the amylases of *Haloarcula* sp. HS. For example, the intracellular amylase of *Haloarcula japonica* presented a molecular mass of 83 kDa [49]; the extracellular amylases of *Haloterrigena tukmenica* and *Haloferax* sp. HA10 showed a molecular weight of 66 kDa [39,47], in *Haloferax mediterranei*, the weight of the enzyme was around 50–58 kDa [26], 60 kDa in *Halorubrum xinjiangense* [46], and 74 kDa in *Natronococcus* sp. Ah-36 [45], while in the *Haloarcula* species, the molecular mass varied from 43 to 70 kDa [27,44]. The physicochemical parameters of the new alpha-amylases, with low isoelectric point and negative net charge (Table 3) also meet the usual characteristic of haloarchaeal enzymes, as reported by other authors. Yan and Wu [43] analyzed the sequences of 88 α-amylases from archaea and observed that amylases from haloarchaea have a highly negatively charged surface, and a higher percentage of acidic residues as a mechanism of adaptation to high salinity. Other authors describe similar features for *H. hispanica*, which has an extracellular amylase with an isoelectric point of 4.2 and a low level of aromatic and hydrophobic residues [27]. In haloarchaea, most studies about amylases focused on extracellular enzymes, given that sometimes no activity was found in the crude cell extract, as was observed in *Haloferax mediterranei* and *Halorubrum xinjiangense* [26,46] or because, as in the case of *Haloterrigena turkmenica*, the amylase activity was quite higher in the supernatant than in the cell extract [39]. With respect to the *Haloarcula* genus, Hutcheon et al. confirmed the overexpression of an extracellular amylase (AmyH) in the mutant strain *Haloarcula hispanica* B3, which was secreted in a folded conformation via the TAT (Twin-Arginine-Translocation) pathway, indicating that it was active in the cytoplasm before the secretion to the media [27]. Additionally, Onodera et al. reported the overexpression of an intracellular amylase (malA), which was not secreted to the media in *Haloarcula japonica* [49]. Based on the above mentioned, it seems that there were diverse amylases with probably different modes of action, which to our knowledge, are not yet deeply elucidated.

Maltose is the main end-product released from the starch hydrolysis by the extracellular and cellular partially purified amylase extracts of *Haloarcula* sp. HS (Table 2). This is the main product of maltogenic α-amylases, like most α-amylases from haloarchaea, e.g., intracellular α-amylase from *Haloarcula japonica* [49] or the extracellular α-amylase from *Haloterrigena tukmenica* [39]. A small proportion of glucose was found in the assay catalyzed by the cell extract. However, it is difficult to predict if it is directly due to the action of the cellular amylases in *Haloarcula* sp. HS or due to the contribution of additional cell-associated enzymes.

With regards to the optimal enzymatic parameters, both cell-associated and extracellular starch-degrading activities exhibited their maximum activities around 60 °C. The low-temperature dependence of the cell-associated amylase, which only loses around 25% of its activity in the temperature range 20–80 °C, is noteworthy. However, it should be considered that this activity might be, as shown in this study, the result of the action of three different amylase enzymes. This fact contributes to broadening the range of optimal temperatures for the cell-associated amylase activity. High retention of enzyme activity over a wide range of temperatures was reported for other partially purified amylases, such as that from *Haloferax* sp. HA10 [47], which showed the highest amylase activity at 55 °C.

The optimal pH values were 7 for the cellular amylase activity, and 5 for the extracellular activity. Additionally, extracellular and cell-associated, amylase activities were extremely halophilic, showing their maximum activities at 25% NaCl. Therefore, it is noteworthy that the cell-associated amylase activity seemed to be more tolerant to changes in salinity, pH, and temperature than the extracellular one (Figure 5). This was probably due to the presence of three different amylases in the cell extract, as was later confirmed in the proteomic analysis. In addition, the high salt and temperature tolerance could be of interest for many industrial applications in which these extreme conditions are needed.

A comparison with the optimal parameters reported for amylases of other related haloarchaea is summarized in Table 4. Most extracellular α-amylases from haloarchaea showed their best activity at temperatures from 45 to 70 °C, pH from 6.5 to 8.7, and in salt concentrations from 1 to 5 M. The extracellular amylase found in *Haloarcula* sp. HS is one of the most halophilic and acidophilic α-amylase described within the haloarchaea group (Table 4).

Additionally, extracellular and cell-associated amylase, activities from *Haloarcula* sp. HS, exhibit a strong inhibition in the presence of the metal chelating agent EDTA (Figure 6). In addition, the analysis of the amylase sequences obtained allowed the identification of the canonical Ca-binding residue in the three amino acidic sequences, as shown in Figure 8, indicating that they must be calcium-dependent. Dependence of calcium is a common feature within haloarchaeal amylases, as was reported for *Haloferax mediterranei*, *Haloarcula hispanica*, *Haloarcula* sp. S-1, *Halorubrum xijiangense*, and [26,27,44,46]. However, some haloarchaeal amylases showed to be resilient to EDTA, indicating no dependence on Ca^2+^, as revealed by the studies on *Haloterrigena turkmenica* and *Haloarcula japonica* [39,49].

Furthermore, both amylase activities showed high stability in most tested surfactants, excepting the anionic detergent SDS. There are few reports on the stability of amylase from haloarchaea on surfactants. In this context, detergent-stable amylases were recently found in *Haloterrigena tukmenica*, *Halorubrum xijiangense*, and *Haloferax* sp. HA10 [39,46,47]. Additionally, a surfactant-stable amylase was characterized from the halophilic bacteria *Nesterenkonia* sp. F [53]. Therefore, to the best of our knowledge, the two detergent-stable amylase activities described in this work entailed the first report on this specific feature of the amylase activity from the *Haloarcula* genus.

Analyzing the sequences of the peptides generated by the tryptic digestion of the partially purified extracellular and cell extracts, it was possible to identify three different amylases in *Haloarcula* sp. HS. One (AMY_HS1) was found both in cells and in the culture medium, while the other two amylases (AMY_HS2 and AMY_HS3) were exclusively found in the cell extract.

Despite the low percentage of sequence identity that the three amylases of *Haloarcula* sp. HS shared (Figure 8), the three enzymes exhibited a high three-dimensional structure homology, with the three typical domains of alpha-amylases of the glycosyl hydrolase GH-13 family (Figure 7) and many of the key conserved residues (Figure 8). For example, the three amino acids (Asp-Glu-Asp), which constituted the active site of alpha-amylases, were identified in α-amylases of haloarchaeal strains, such as *Halogeometricum borinquense* [30] and *Haloterrigena turkmenica* [39]. These residues were conserved in all alpha-amylases of the GH-13 family of many different origins, which were compiled in the Carbohydrate-Active enZYmes (CAZy) database [54].

The calcium-binding domain located between the 3rd beta-strand and the 3rd alpha-helix contained an asparagine amino acid found in the three amylases of *Haloarcula* sp. HS (Figure 8), as was described for other haloarchaeal amylases [30] and many other α-amylases, which were calcium-dependent metalloenzymes [55]. In some cases, more than one Ca-binding domain was described, as in *H. orenii* [56]. Additional conserved amino acids reported in alpha-amylases, which help to stabilize the structure or the binding of the substrate [5], were found in the three amylases of *Haloarcula* sp. HS (Figure 8).

Most available archaeal amylase sequences were from the thermophilic archaea and could work at very high temperatures, which was of great industrial interest. The amylases identified from the Odiel Marshlands were medium or highly thermotolerant, in addition to being extremely halophilic. The α-amylases from hyperthermophilic archaea were closely related to plant amylases [57]. However, as new potential α-amylases from the halophilic archaea were identified, evident differences were observed with the sequences of their known hyperthermophilic counterparts. Unfortunately, most of those halophilic amylolytic enzymes were only putative proteins from genome sequencing projects [58], or their complete sequences were not available [26,44,46], making it difficult to establish accurate phylogenetic relationships. In addition, an enormous diversity was observed among the amylases characterized and sequenced from a member of the *Halobacteriaceae* family, which showed similarities with marine bacteria, fungal, or even animal sources [59]. Therefore, more insightful biochemical characterization studies are needed to reveal the exact features of these amylolytic enzymes from haloarchaea.

Although several copies of alpha-amylases appear in the sequenced genomes of haloarchaea, most studies are focused on the extracellular ones. The role of the extracellular amylase in haloarchaea is well-established, as it allows the conversion of starch, produced by the marine plankton, into simple sugars that could be incorporated into the cell and used as a carbon source [60]. Nonetheless, the function of the intracellular amylases is less understood, not only in haloarchaea but also in other heterotrophic microorganisms [61]. Most intracellular amylases of haloarchaea were assigned by sequence homology without a functional characterization, with few exceptions, like the intracellular α-amylase from *H. japonica*, whose activity is well-studied [49]. Further insight is necessary to complete the characterization of haloarchaeal amylases, to understand their role in archaeal metabolism, and to evaluate their biotechnological applications.

## 5. Conclusions

The detailed biochemical characterization of the cell-associated and the extracellular amylase activities from the new isolated strain *Haloarcula* sp. HS revealed that both are active at high salinity conditions and at considerably high temperatures. These features, joined to their stability under a wide range of surfactants, make them suitable for industrial applications. The proteomic analysis showed that three different cell-associated enzymes, one of which was also found in the extracellular medium, might be responsible for the amylase activities. The three proteins conserve the consensus domains and residues of the α-amylase family. Further studies aim to decipher the function of a hypothetical ancestral gene and to increase our understanding of the biochemical behavior of these polyextremophilic enzymes. Developing new techniques for high-scale production in the industry is also needed.

## Figures and Tables

**Table 1:** Sequences of the primers employed for the amylase encoding genes amplification.

Primers	Forward (5′-3′)	Reverse (5′-3′)
*AMY_HS1*	ACCGGCAGTAAGCAGGCGTCTC	GGCGGCGTCCCAGCGAATACC
	GGCTCGTCGGGCTGAAGGACC	CCCTCTCGCTCGTAGACGTACAGGTC
*AMY_HS2*	CGTCGGCGAATCGGTCGAACT	GTCGCGTTTCCGGTTCCACTGTC
	GGAACGCGACAGTGGAACCGGA	CGAAGTGCAGAACGACCACGAGCG
*AMY_HS3*	GGAGACGGCCCGGTCGAACA	CGCGTCGAAGGGCGATTC
	GCCGGCGATAGCGACGAAT	TCGTACGGGATTCGGAGGAGG

Primer sets used for PCR amplification of the three amylase coding genes found in *Haloarcula* sp. HS. For each gene (*AMY_HS1*, *AMY_HS2*, and *AMY_HS3*), two pairs of primers were designed on the basis of the sequences of the peptides obtained by proteomics.

**Table 2:** Percentage of the different products obtained from starch hydrolysis.

	Dextrins (%)	Maltose (%)	Glucose (%)	Starch (%)
Cell-associated amylase	ND	86.1 ± 3.6	6.6 ± 0.8	7.3 ± 2.9
Extracellular amylase	18.5 ± 1.1	79.7 ± 1.3	ND	1.7 ± 0.2
Commercial α-amylase	20.8 ± 3.1	73.8 ± 3.4	ND	5.4 ± 0.3

Comparison of the products obtained from the hydrolysis of starch by cell-associated or extracellular partially purified extracts of *Haloarcula* sp. HS and a commercial α-amylase. The percentage (%) of dextrins, maltose, and glucose produced and the remaining starch are indicated as the mean of three replicates with the corresponding standard deviation. ND, not detected.

**Table 3:** Physicochemical properties of the amylase enzymes from *Haloarcula* sp. HS.

Name	N	MW (kDa)	IP	Z	GRAVY	Aliph. Index
AMY_HS1	393	43.70	4.27	−35.577	−0.518	72.72
AMY_HS2	639	70.16	4.43	−66.227	−0.382	73.43
AMY_HS3	549	60.02	4.37	−59.604	−0.463	71.89

Main physicochemical characteristics of the amylase enzymes found in the *Haloarcula* sp. HS strain. N, number of nucleotides; MW, molecular weight; IP, theoretical isoelectric point; Z, net charge; GRAVY (Grand average of hydropathicity), mean of the hydropathy index of each amino acid residue. The aliphatic index stands for the relative volume occupied by the aliphatic side chains.

**Table 4:** Optimal parameters reported for α-amylase activity in haloarchaea.

Microorganism	Enzyme	NaCl (M)	pH	Tª (°C)	Ref.
*Haloarcula* sp. HS	Cellular α-amylase	2.6	7	50	This study
*H. japonica*	Intracellular α-amylase	2.6	6.5	45	[49]
*Haloarcula* sp. HS	Extracellular α-amylase	5	5	60	This study
*Halococcus* GUVSC8	Extracellular α-amylase	2	6	45	[48]
*Haloarcula* sp. S-1	Extracellular α-amylase	4.3	7	50	[44]
*H. hispanica* B3	Extracellular α-amylase	4–5	6.5	50	[27]
*H. hispanica* 2TK2	Extracellular α-amylase	5	6.9	52	[50]
*H. xinjiangense*	Extracellular α-amylase	4	8.5	70	[46]
*Haloferax* sp. HA10	Extracellular α-amylase	1	6	55	[47]
*H. mediterranei*	Extracellular α-amylase	3	7–8	50–60	[26]
*H.turkmenica*	Extracellular α-amylase	2	8.5	55	[39]
*N.amylolyticus*	Extracellular α-amylase	2.5	8.7	55	[45]

## Data Availability

All DNA and protein sequences of the studied enzymes are included as Supplementary Materials; other information is available upon request.

## Supplementary Materials

The following are available online at https://www.mdpi.com/article/10.3390/biology10040337/s1. Figure S1: Full length of the 16S rRNA encoding gene from *Haloarcula* sp. HS. Figure S2: Complete original polyacrylamide gels. Figure S3: Predicted structures of the three amylase sequences identified in *Haloarcula* sp. HS. Figure S4: Multiple alignments of the amino acid sequence of the extracellular amylase identified in *Haloarcula* sp. HS (AMY_HS1). Figure S5: Multiple alignments of the amino acid sequence of the cell-associated amylase from *Haloarcula* sp. HS (AMY_HS2). Figure S6: Multiple alignments of the amino acid sequence of the cell-associated amylase from *Haloarcula* sp. HS (AMY_HS3).
