# Supplementary material for: Biochemical Characterization of the Amylase Activity from the New Haloarchaeal Strain Haloarcula sp. HS Isolated in the Odiel Marshlands
Source: Biology (Basel). 2021 Apr 16;10(4):337. doi: 10.3390/biology10040337 (PMC8073556; doi:10.3390/biology10040337)
Supplement: Supplementary file 1 [file biology-10-00337-s001.zip › Supplementary Material-V3/Figure S1_16S_coding_gene_sequence.docx]

GGAATCGATTAGCCCTGCTAGTCGCACGGGTCTTAGACTCCGTAGGCATATAGCTCAGTAACACGTGGCCAAACTACCCTACAGACCGCGATAACCTCGGGAAACTGAGGCCAATAGCGGATATAACTCTCAGGCTGGAGTGCCGAGAGTTAGAAACGTTCCGGCGCTGTAGGATGTGGCTGCGGCCGATTAGGTAGATGGTGGGGTAACGGCCCACCATGCCGATAATCGGTACGGGTTGTTGGAGAGCAAGAACCCGGAGACGGTATCTGAGACAAGATACCGGGCCCTACGGGGCGCAGCAGGCGGGAAACCTTTACACTGCACGACAGTGCGATAGGGGGACTCCGAGTGTGAGGGCATATAGCCCTCGCTTTTCTGTACCGTAAGGTGGTACAGGAACAAGGACTGGGCAAGACCGGTGCCAGCCGCCGCGGTAATACCGGCAGTCCAAGTGATGGCCGATATTATTGGGCCTAAAGCGTCCGTAGCTTGCTGTGTAAGTCCGTTGGGAAATCGACCCGCTCAACGCGTCGGCGTCCAGCGGAAACTGTCCGGCTTGGGGCCGGAAGACTTGGGGGGTACGTCCGGGGTAGGAGTGAAATCCTGTAATCCTGGACGGACCACCAATGGGGAAACCACCTTGAGAAGCCGGACCCGACGGTGAGGGACGAAAGCCAGGGTCTCGAACCGGATTAGATACCCGGGTAGTCCTAGCTGTAAACGATGCTCGCTAGGTGTGCCGTAGGCCACGAGCATGCGATGCGCCGTAGGGAAGCCGAGAAGCGAGCCGCCTGGGAAGTACGTCTGCAAGGATGAAACTTAAAGGAATTGGCGGGGGAGCACCACAACCGGAGGAGCCTGCGGTTTAATTGGACTCAACGCCGGAAATCTCACCGGTCCCGACAGTAGTAATGACGGTCAGGTTGACGACTTTACCCGACGCTACTGAGAGGAGGTGCATGGCCGCCGTCAGCTCGTACCGTGAGGCGTCCTGTTAAGTCAGGCAACGAGCGAGACCCGCACTTCTAGTTGCCAGCAATACCCTTGAGGTAGTTGGGTACCCTAGGAGGACTGCCGCTGCTAAAGCGGAGGAAGGAACGGGCAACGGTAGGTCAGTATGCCCCGAATGGACCGGGCAACACGCGGGCTACAATGGCTCTGACAGTGGGATGCAACGCCGAGAGGCGACGCTAATCTCCAAACGGAGTCGTAGTTCGGATTGCGGGCTGAAACCCGCCCGCATGAAGCTGGATTCGGTAGTAATCGCGTGTCAGAAGCGCGCGGTGAATACGTCCCTGCTCCTTGCACACACCGCCCGTCAAAGCACCCGAGTGGGGTCCGGATGAGGCCGTCATGCGACGGTCGAATCCT

**Figure S1.** Full length of the 16S rRNA encoding gene from *Haloarcula* sp. HS, amplified with the archaeal specific primers 21F (5’-TTCCGGTTGATCCTGCCGGA-3’) and 1492R (5’-GGTTACCTTGTTACGACTT-3’). Polymerase chain reactions (PCR) were performed as indicated in Materials and Methods.
