# Supplementary material for: Biochemical Characterization of the Amylase Activity from the New Haloarchaeal Strain Haloarcula sp. HS Isolated in the Odiel Marshlands
Source: Biology (Basel). 2021 Apr 16;10(4):337. doi: 10.3390/biology10040337 (PMC8073556; doi:10.3390/biology10040337)
Supplement: Supplementary file 1 [file biology-10-00337-s001.zip › Supplementary Material-V3/Figure S2_Polyacrylamide_gels.docx]

**Figure S2.** Polyacrylamide gels corresponding to the whole gels of Figure 4. The cropped lanes with their respective numbers are denoted by dashed boxes for Coomassie and Lugol staining.
