# Supplementary material for: Biochemical Characterization of the Amylase Activity from the New Haloarchaeal Strain Haloarcula sp. HS Isolated in the Odiel Marshlands
Source: Biology (Basel). 2021 Apr 16;10(4):337. doi: 10.3390/biology10040337 (PMC8073556; doi:10.3390/biology10040337)
Supplement: Supplementary file 1 [file biology-10-00337-s001.zip › Supplementary Material-V3/Figure S3_Amylase_sequences and structures.docx]

**Figure S3.** Predicted structures of the three amylase sequences identified in *Haloarcula* sp. HS; A: extracellular amylase, AMY_HS1; B: cell-associated amylase, AMY_HS2; C: cell-associated amylase Amy_HS3. 2D structures were made using the NetSurfP software. Helixes are represented by ribbons and strands by arrows. The red color indicates surface exposition, while the blue color shows buried surfaces.
