# Supplementary material for: Biochemical Characterization of the Amylase Activity from the New Haloarchaeal Strain Haloarcula sp. HS Isolated in the Odiel Marshlands
Source: Biology (Basel). 2021 Apr 16;10(4):337. doi: 10.3390/biology10040337 (PMC8073556; doi:10.3390/biology10040337)
Supplement: Supplementary file 1 [file biology-10-00337-s001.zip › Supplementary Material-V3/Figure S4_Amylase_alignments_HS1.docx]

**Figure S4.** Multiple alignments of the amino acid sequence of the extracellular amylase identified in *Haloarcula* sp. HS (AMY_HS1) with alpha-amylases from different haloarchaea. The sequences are ordered from the top to the bottom with decreasing degree of identity: *Haloarcula hispanica* N601 (WP_014040218.1), *Halomicroarcula salina* (WP_162411878.1), *Halapricum salinum* (WP_049992672.1), and *Haloterrigena turkmenica* (WP_012943282.1). Purple stars highlight the catalytic triad (Asp-Glu-Asp), blue star denotes the canonical calcium-binding site and black stars point other essential residues for enzyme structure. Residues corresponding to the TAT motif are denoted with black boxes and the processing site is indicated by a black dashed double arrow. The gradient from red to white in the background indicates the degree of conservation, with white 100%. Secondary structures, helixes, and strands are denoted by blue and red boxes, respectively, matching with the 3D protein models shown in Figure 8.
