# Supplementary material for: Biochemical Characterization of the Amylase Activity from the New Haloarchaeal Strain Haloarcula sp. HS Isolated in the Odiel Marshlands
Source: Biology (Basel). 2021 Apr 16;10(4):337. doi: 10.3390/biology10040337 (PMC8073556; doi:10.3390/biology10040337)
Supplement: Supplementary file 1 [file biology-10-00337-s001.zip › Supplementary Material-V3/Figure S6_Amylase aligments_HS3.docx]

**Figure S6.** Multiple alignments of the amino acid sequence of the cell-associated amylase from *Haloarcula* sp. HS (AMY_HS3) with alpha-amylases from different haloarchaea. The sequences are ordered from the top to the bottom with decreasing degree of identity: *Haloarcula hispanica* N601 (WP_023843400.1), *Halomicroarcula salina* (WP_162414602.1), *Haloferax mediterranei* (WP_004056521.1), and *Halogeometricum limi* (WP_089883475.1). Purple stars highlight the catalytic triad (Asp-Glu-Asp), blue star denotes the canonical calcium-binding site and black stars point other essential residues for enzyme structure. The gradient from red to white in the background indicates the degree of conservation, with white 100%. Secondary structures, helixes, and strands are denoted by blue and red boxes, respectively, matching with the 3D protein models shown in Figure 8.
